# Cognitive fatigue and cortical-striatal network in old age

**DOI:** 10.18632/aging.101915

**Published:** 2019-04-17

**Authors:** Ping Ren, Andrew J. Anderson, Kelsey McDermott, Timothy M. Baran, Feng Lin

**Affiliations:** 1 School of Nursing, University of Rochester Medical Center, Rochester, NY 14623, USA; 2 Department of Neuroscience, University of Rochester Medical Center, Rochester, NY 14623, USA; 3 Department of Biomedical Engineering, University of Rochester, Rochester, NY 14623, USA; 4 Department of Imaging Sciences, University of Rochester Medical Center, Rochester, NY 14623, USA; 5 Department of Psychiatry, University of Rochester Medical Center, Rochester, NY 14623, USA; 6 Department of Neurology, University of Rochester Medical Center, Rochester, NY 14623, USA; 7 Department of Brain and Cognitive Science, University of Rochester, Rochester, NY 14623, USA

**Keywords:** cognitive fatigue, functional MRI, functional connectivity, striatum, Posterior-Anterior Shifting in Aging

## Abstract

Cognitive fatigue (CF) is among the most common and disturbing aging symptoms, and substantially interferes with activities demanding sustained mental effort. Here we examined the relationship between the cortical-striatal network and CF (assessed by the 18-item visual analogue scale) when a group of cognitively and physically healthy older adults participated in a 30-minute cognitively fatiguing task-related fMRI experiment. We also explored whether CF would interfere with the “Posterior-Anterior Shifting in Aging” (PASA) phenomenon, an aging-associated neural reliance on frontal regions to support cognitive capacity. We revealed that decreased connectivity strength of the cortical-striatal network over the course of the task was related to higher CF. Correlation between CF and the cortical-striatal network was more robust in anterior relative to posterior components. Moreover, a positive relationship between reliance on the anterior part of the cortical-striatal network and cognitive performance only existed among older adults experiencing low CF. These findings suggest a crucial role of the cortical-striatal network, especially the anterior component, in linking to CF. The PASA phenomenon may only be applicable to older adults without vulnerability to CF.

## Introduction

Fatigue is one of the most common and disturbing symptoms in aging, characterized by increased ease of becoming tired, lack of energy, and failure to sustain attention [1–3]. Physical fatigue refers to the muscles’ inability to maintain optimal physical performance when engaging in physically demanding tasks, while cognitive fatigue (CF) refers to decrement in mental effort or self-motivation when engaging in cognitively demanding tasks [1,3]. Compared with physical fatigue, CF is equally problematic for aging populations’ well-being, but less studied. Older adults bear a high risk of adverse consequences from CF in multiple domains, including cognitive decline, degraded daily performance (e.g., driving), and reduced social interaction [4–8].

Multi-modal PET and fMRI studies indicate that the deficient neuromodulation seen in CF may relate to increased neuronal noise from dysfunction of catecholamine (e.g., dopamine) transportation between the striatum and prefrontal cortex (PFC) [9,10]. Patients with multiple sclerosis (MS) are the most frequently examined model for understanding neural correlates of CF. Both task-related and resting-state fMRI studies have shown that CF is associated with abnormal activation or connectivity strength seeded in the basal ganglia and PFC [11–13]. In line with these findings, our previous work reported that higher frontal-striatal connectivity was related to lower perceived CF in patients with mild cognitive impairment [8]. Overall, converging evidence from disease models suggests the frontal-striatal circuit is crucial for understanding CF [14].

There have been limited studies examining neural mechanisms of CF in normal aging, or how cognitive capacity is affected by CF-associated neural networks when older adults perform cognitively fatiguing tasks. These aspects may help explain the commonly observed detrimental effects of CF on everyday functions with cognitive demand [15]. Older people tend to recruit additional brain regions or overuse some regions that are not commonly used in younger age to support performance during cognitively demanding tasks [16,17]. Aging-associated CF may therefore be linked to regions beyond the frontal-striatal circuit. Particularly, the Posterior-Anterior Shift in Aging (PASA) phenomenon, defined as the enhanced activation and connectivity in frontal regions with decreased activity in posterior regions, may be relevant in the current context [18–20]. The PASA model has been mainly used to explain the change in brain patterns involved in the cognitive aging process, as well as the cognitive maintenance due to PASA. It is unclear whether there is a direct linkage between the PASA phenomenon and vulnerability to the experience of CF, or whether the relationship between cognitive performance during a cognitively fatiguing task and PASA may be affected by CF. Examining the PASA model within the cortical-striatal network may help understand aging-associated CF, as well as the relationship between CF and cognitive capacity during cognitively fatiguing tasks.

**Figure 1:**
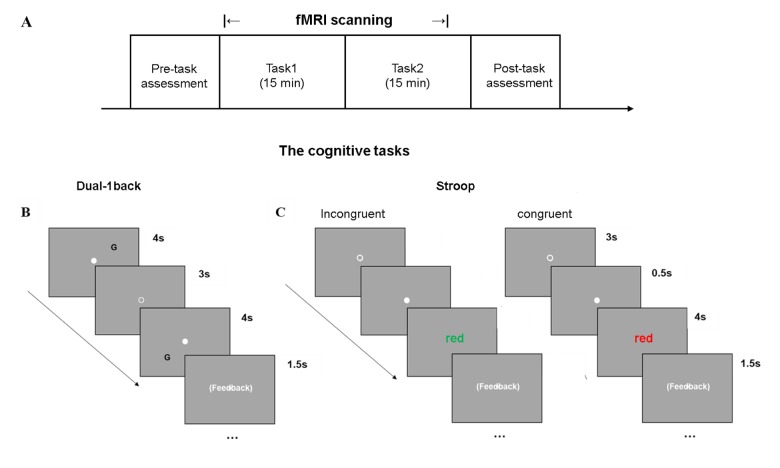
**Cognitively demanding tasks used to induce CF.** (**A**) The experimental paradigm included two cognitively fatiguing tasks during fMRI scanning, in a random order, across subjects. (**B**) Dual-1 back task was a letter-location task. (**C**) The Color-word Stroop task consisted of congruent and incongruent conditions on color.

In the current study, we address the relationship between the cortical-striatal network and experience of CF, as well as how this relationship influences cognitive performance by examining the PASA phenomenon in the involved neural circuit. To induce CF in older adults, we administer a mixed set of executive function tasks (Dual 1-back and Color-word of the Stroop task) during fMRI scanning (see Figure 1). Also, we employ a group of cognitively and physically healthy older adults to eliminate the potential confounding effect from chronic diseases for CF. Our aims are to: 1) identify the cortical-striatal network related to the experience of CF when older adults perform cognitively fatiguing tasks; 2) compare the difference between anterior and posterior cortical-striatal network in relation to the experienced CF; 3) explore whether the experience of CF affects the known relationship between the PASA model and cognitive capacity.

## RESULTS

Sample used here was from 46 subjects (6 subjects were removed due to the head motion). There was no difference in the demographic and behavioral data between those who were removed and remained in the analysis. Based on the change of CF scores as we described above, two subgroups with distinct CF sensitivity were defined as low-CF (n = 12) and high-CF (n = 14) in initial analysis. Comparisons of the two subgroups on sample characteristics are presented in Supplementary Table 1.

### Demographic and behavioral data analysis

**Figure 2:**
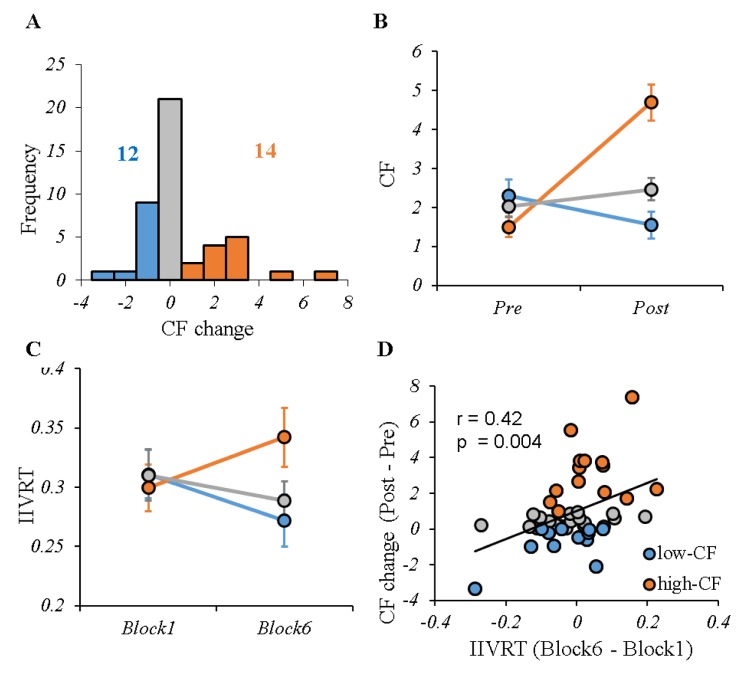
**CF profile for the entire sample.** (**A**) The distribution of CF change (post-pre) induced by cognitive tasks during fMRI scanning. According to different perceived fatigue, two groups were selected: low-CF subgroup (12 subjects with CF change ≤ 0, shown in blue) and high-CF subgroup (14 subjects with CF change ≥ 1, shown in red). In addition, there were 20 subjects outside the high- or low-CF subgroups (shown in grey). (**B**) The VAS fatigue score before and after performing cognitive tasks for each group, showing significant difference between high- and low-CF subgroup. (**C**) The IIVRT in Block1 and Block6 for each group showed the performance change with time by CF subgroups. There were significant different trends in IIVRT change between high- and low-CF subgroup. (**D**) Higher CF was significantly associated with larger IIVRT change (worse cognitive performance). Note: CF, cognitive fatigue; IIVRT, intra-individual variability of reaction time. low-CF subgroup: blue; high-CF subgroup: red; others: gray.

The sample characteristics, as well as their correlations with CF in the entire sample are shown in Table 1. There was a significant subgroup (high- vs. low-CF) by block (1 vs. 6) interaction on fatigue score from VAS (F(1, 24) = 48.67, p < 0.001) (Figure 2B). Figure 2C shows a significant CF subgroup (low- vs. high-CF) by task block (1 vs. 6) interaction on IIVRT (F(1, 24) = 4.81, p = 0.038). Consistently, IIVRT analysis showed the task performance became worse in individuals with higher CF, supporting our approach of low- and high-CF group definition. IIVRT was positively correlated with CF scores (r = 0.42, p = 0.004), but not MOCA (r = -0.11, p = 0.49) (Figure 2D). Additionally, there was neither significant interacting effect between task type and block (F(1, 44) = 0.23, p = 0.64) on IIVRT, nor main effect of task type on IIVRT (F(1, 44) = 1.03, p = 0.32).

**Table 1:** Demographics and clinical characteristics (n = 46).

	**Descriptive**	**Correlation (r) with CF-pre (p value)**	**Correlation with CF change (p value)**
**Age, Mean (SD)**	71.52 (±5.27)	-.015 (.92)	.15 (.33)
**Male, n (%)**	15 (32.6)	-	-
**Years of education, Mean (SD)**	16.76 (±2.84)	.03 (.84)	-.16 (.28)
**MOCA, Mean (SD)**	27.85 (±1.69)	-.12 (.42)	.28 (.056)
**CF-pre, Mean (SD)**	1.94 (±1.25)	-	**-.35 (.016)**
**CF change, Mean (SD)**	.96 (±1.89)	**-.35 (.016)**	**-**
**IIVRT change, Mean (SD)**	-.0065 (±.10)	-.09 (.55)	**.42 (.004)**
**Brain atrophy, Mean (SD)**	.34 (±.034)	-.14 (.34)	-.20 (.18)
**Executive abilities, Mean (SD)**	0.56 (±0.40)	-.21 (.16)	.14 (.36)

Note. MOCA, Montreal Cognitive Assessment; CF, cognitive fatigue; IIVRT, intra-individual variability of reaction time; SD, standard deviation.

### Cortical-striatal network in low- and high-CF subgroups

**Figure 3:**
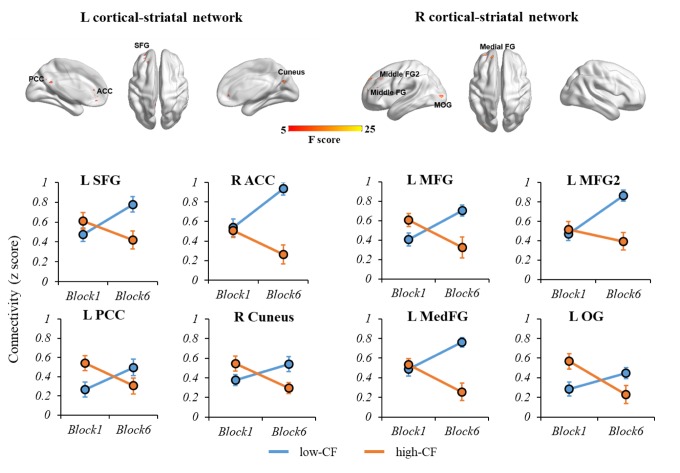
**The interaction effects of CF subgroup and task block on left and right cortical-striatal network connectivity.** For the left cortical-striatal network, there were significant differences of connectivity change in L SFG, R ACC, L PCC and R Cuneus. For the right cortical-striatal network, there were significant differences of connectivity change in L MFG, L MFG2, L MedFG and L OG. During the fatiguing tasks, the connectivity strength of cortical-striatal network increased in low-CF group, while deceased in high-CF group. Note: L, left; R, right; SFG, superior frontal gyrus; ACC, anterior cingulate cortex; PCC, posterior cingulate cortex; MedFG, medial frontal gyrus, MFG, middle frontal gyrus; OG, occipital gyrus. CF, cognitive fatigue.

Examining the interaction effects of CF subgroup (low- vs. high-CF) and task block (1 vs. 6), we identified four significant regions in the left cortical-striatal network: left superior frontal gyrus (SFG), right anterior cingulate cortex (ACC), left posterior cingulate cortex (PCC), and right cuneus, and four significant regions in the right cortical-striatal network: left medial frontal gyrus (MedFG), left middle frontal gyrus (MFG), left MFG2, and left occipital gyrus (OG). Table 2 provides a description on each region. Figure 3 displays the relationship between functional connectivity of each cortical-striatal network across two task blocks by CF subgroup.

**Table 2:** Overview of regions where cortical-striatal functional connectivity was associated with CF (n = 46).

**Region**	**Peak F**	**Clusters**	**MNI coordinates**
**Left striatum network** CF subgroup*task block			
Left PCC	17.3	33	-3, -51, 30
Left SFG	16.5	24	-21, 39, 39
Right ACC	24.2	27	12, 36, 0
Right Cuneus	21.3	16	15, -72, 27
**Right striatum network**			
CF subgroup*task block			
Left MedFG	24.4	35	-12, 33, 42
Left MFG	19.2	17	-48, 36, 18
Left MFG2	16.1	46	-33, 51, 15
Left OG	23.6	17	-33, -90, 3

Significant brain regions were determined using repeated measures ANOVA of CF subgroup (high- vs. low-CF) and task block (block 1 vs. block 6) (p<0.05, GRF correction). MNI, Montreal Neurological Institute. PCC, posterior cingulate cortex; SFG, superior frontal gyrus; ACC, anterior cingulate cortex; MedFG, medial frontal gyrus; MFG, middle frontal gyrus; OG, occipital gyrus; L, left; R, right.

### Correlations between cortical-striatal network and CF

**Figure 4:**
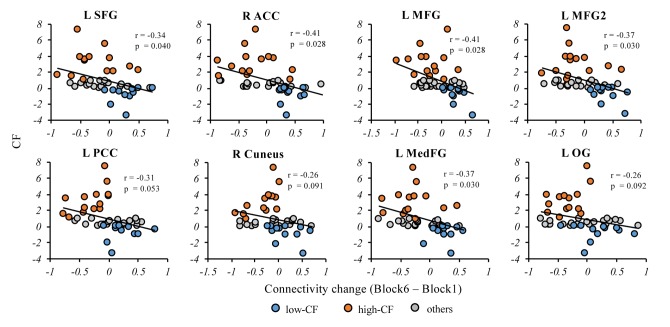
**Correlations between connectivity change and CF in the entire sample.** In the cortical-striatal network, the partial correlation analysis showed CF change was significantly correlated with the anterior regions, including L SFG, R ACC, L MFG, LMFG2 and L MedFG. The CF change showed negative correlation trend with the posterior regions but not significant, including L PCC, R Cuneus and L OG. Note: L, left; R, right; SFG, superior frontal gyrus; ACC, anterior cingulate cortex; PCC, posterior cingulate cortex; MedFG, medial frontal gyrus, MFG, middle frontal gyrus; OG, occipital gyrus. p values here are FDR-corrected across all regions. CF, cognitive fatigue; IIVRT, intra-individual variability of reaction time.

To examine correlations between the cortical-striatal network and CF in the entire sample, partial correlation was applied to each brain region we found above controlling for age, education and whole-brain gray matter volume. Greater CF was significantly related to less increase in functional connectivity between striatum and the four frontal regions identified above, but not posterior regions, for False Discovery Rate (FDR)-corrected p < 0.05 (Figure 4). We also examined correlations between striatal network and CF for Stroop and 1-back tasks separately, and found similar patterns (Supplementary Figure 2).

### Anterior vs. posterior cortical-striatal network analysis

**Figure 5:**
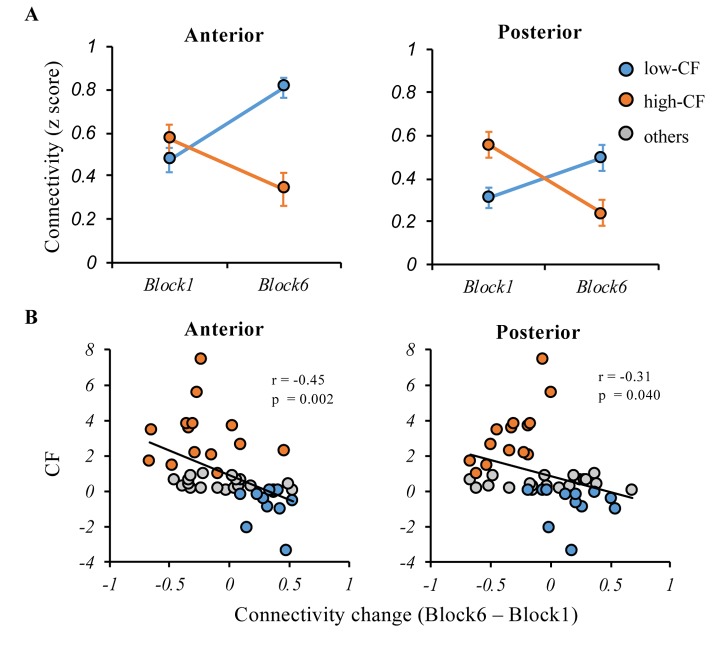
**Relationships between the anterior and posterior cortical-striatal network functional connectivity and CF.** (**A**) During the cognitively fatiguing tasks, both of the anterior and posterior network showed significant interaction effects between subgroup (high- vs. low-CF) and block (1 vs. 6), that connectivity strength increased in low-CF group while decreased in high-CF group (**B**) Although CF was negatively associated with change of anterior or posterior connectivity, the negative relationship was more robust in anterior cortical-striatal network. Note: CF, cognitive fatigue; IIVRT, intra-individual variability of reaction time.

To examine the relationship between anterior and posterior cortical-striatal network and CF, we calculated the functional connectivity for anterior and posterior cortical-striatal networks separately. There was a significant subgroup (high- vs. low-CF) by block (1 vs. 6) interaction on the functional connectivity for both anterior (F_(1, 24)_ = 16.21, p < 0.001) and posterior network (F_(1, 24)_ = 8.34, p = 0.001) (Figure 5A). Using the entire sample, the CF change was significantly correlated with both change of functional connectivity in the anterior network (r = -0.45, p = 0.002), and posterior network (r = -0.40, p = 0.04). Notably, the correlation between CF change and anterior network was more robust relative to the posterior network (Figure 5B).

### Posterior-anterior shift, CF and cognitive function

**Figure 6:**
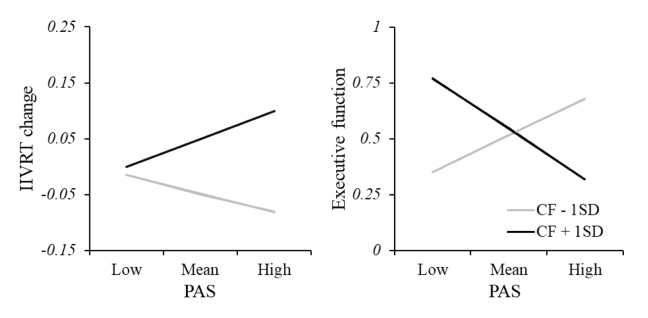
**Moderation analysis of relationship between PAS and cognitive function.** There was a significant interacting effect between CF and PAS in predicting task performance, showing greater PAS was related to better cognitive performance (smaller IIVRT) in the low-CF subgroup while worse cognitive performance (larger IIVRT) in the high-CF subgroup. Consistently, CF also significantly interacted with PAS in predicting executive function. Note: CF, cognitive fatigue; IIVRT, intra-individual variability of reaction time. PAS, posterior-anterior shifting.

PAS was calculated to measure the posterior-anterior shift over the course of cognitively fatiguing tasks. Moderation analysis was applied to examine the influence of CF level on the relationship between PAS and cognitive function (Figure 6). CF significantly interacted with PAS in predicting task performance during the fatiguing tasks (R^2^ = 0.29, F(6, 39) = 2.62, p = 0.031); greater PAS was related to better cognitive performance in the low-CF subgroup while worse cognitive performance in the high-CF subgroup. CF also significantly interacted with PAS in predicting executive function (R^2^ = 0.32, F(6, 39) = 3.06, p = 0.015). We also calculated the moderation models for anterior and posterior cortical-striatal network separately. Of note, there was a significant moderating effect of CF change on anterior, but not posterior, network and IIVRT change/executive function (see Supplementary Figure 3).

## DISCUSSION

In the current study, we examined the association of the cortical-striatal network with the experience of CF when older adults performed cognitively fatiguing tasks. By calculating the interaction between time blocks of the task and groups with high vs. low CF, we revealed multiple regions in the cortical-striatal network where decreased connectivity strength over the course of the task was related to higher level of CF. The involved regions included multiple anterior (i.e., left SFG, right ACC, left MFG, left MFG2, and left MedFG) and posterior regions (i.e., left OG, left PCC, and right cuneus). After averaging connectivity strengths for frontal-striatal vs. posterior-striatal networks separately, both anterior and posterior networks were significantly related to CF, while the anterior network’s correlation was more robust. Moreover, the CF level moderated the association of the PAS score with cognitive performance. That is, increased posterior-anterior shift with better cognitive performance only occurred for older adults experiencing low CF, while the relationship was opposite in those with high CF. The validity of the model was also extended to predict general executive function that was not directly assessed in the tasks. The findings suggest that the change of cortical-striatal network associated with CF may help explain older adults’ cognitive inefficiency in cognitively fatiguing tasks.

In the current study, we first selected two extreme subgroups (low- and high- CF) based on individual’s perceived vulnerability to CF. Both self-report CF and task performance (IIVRT) showed similar pattern across the two groups. The positive correlation between CF and IIVRT indicated that increased CF induced more behavioral variation and worse cognitive performance during the tasks. Consistently, previous studies have also reported that individuals with CF showed disrupted cognitive function, and declines in task performances [21,22]. For the first time, however, a direct association between self-report CF and cognitive performance in the cognitively fatiguing task was identified. Additionally, the current study used two different tasks to explore fatigue related neural substrate, avoiding task specific results. Although the two tasks were slightly different in the task paradigm (an extra 0.5 second-fixation in Stroop), the data analysis in supplements showed that they induced comparable CF (Supplementary Figures 1 and 2).

We found multiple regions within the cortical-striatal network that showed decreased connectivity with increasing experience of fatigue. In the literature, these regions either attend the regulation of working memory or inhibition (e.g., frontal regions) [23], or support motivation (e.g., striatum, ACC) [24]. The experience of CF is most relevant to the decreased strength of networks involving these regions, which implicates the potential influence of CF on cognitive efficiency or motivation. However, we cannot fully differentiate these two circumstances based on the current design. Furthermore, when comparing the posterior vs. anterior brain regions’ relationship with CF, both were related to CF, while the relationship was more robust for the anterior regions. This finding suggests that while anterior regions play a dominant role in supporting cognitive efficiency in old age [18,25], these regions are also more vulnerable to the effect of CF. Notably, we did not find any regions with increased connectivity with the experience of CF. This suggests the compensatory mechanism implied in the cognitive aging literature [26] may be conditional. For the subset of older adults who are particularly vulnerable to fatigue in any cognitive tasks, they may exhaust potential compensatory pathways.

More importantly, we found that CF moderated the relationship between PAS and cognitive performance both inside and outside the cognitively fatiguing tasks. The PASA phenomenon – greater reliance on anterior, relative to posterior regions supporting better cognitive performance – only exists in those experiencing low CF. Conversely, in older adults who are vulnerable to CF, greater connection strength between anterior regions and the striatum may reflect more neural noise and consequently, worse cognitive performance. Among these older adults, reserved neural resources from the posterior region [17] may provide limited supports for their cognitive performance, and the PASA phenomenon may not be applicable. However, whether the reserved posterior regions support cognitive performance among individuals vulnerable to CF requires a comparison with younger adults. Regardless, existing cognitive aging studies have found contradictory results regarding the direction of relationships between anterior regions’ activities and cognitive performance [27–30]. The findings here may help explain this inconsistency by incorporating understanding of individuals’ vulnerability to CF during tasks requiring sustained mental efforts.

There are several limitations in the current study. First, we used a seed-based analysis instead of whole-brain exploration, analyzing neural networks seeded in the striatum. Since previous studies have reported multiple brain regions that are involved in CF (e.g. hypothalamic-brainstem networks) [31,32], other relevant brain networks need to be considered in future research. Second, we only included two cognitive tasks (Stroop and Dual 1-back) in the current study to induce acute CF. Although CF was significantly linked to performance in the tasks, these tasks are still different from everyday activities with cognitive demands. Future studies may consider or develop ecologically validated cognitively fatiguing tasks. Additionally, the number of low- and high-CF subjects within each CF-manipulation task (Stroop or Dual 1-back) was relatively small, which may result in slightly different patterns of CF or IIVRT in these individual tasks (Supplementary Figures 1 and 2) vs. across the tasks (Figures 2-4). Lastly, since the current study did not include a younger comparison, a case-control study is needed to determine whether the CF related neural mechanisms revealed in the current study is constrained by the aging effect.

In summary, our findings support the importance of cortical-striatal networks underpinning the experience of CF in old age. The distinct pattern of anterior vs. posterior brain regions in linking to CF may further explain the mechanism underlying CF among older adults. Moreover, the reliance on anterior regions to support cognitive performance in old age may be disrupted by the experience of or vulnerability to CF.

## METHODS

### Participants

Fifty-two older adults with normal cognition, indexed by Montreal Cognitive Assessment ≥ 26 and Rey Auditory Verbal Learning Test delayed recall ≥6, were recruited in the current project. Participants were required to have intact capacity to give consent (indexed by intact score at UCSD Brief Assessment of Capacity to Consent, UBACC), have adequate visual and auditory acuity for testing by self-report, be free from major depression (indexed by Geriatric Depression Scale-15 item score <6), sleep disorder (indexed by Pittsburg Sleep Quality Inventory global sleep quality score < 14), and chronic fatigue (indexed by 20-item Multidimensional Fatigue Inventory general fatigue subscale < 20), be ≥60 years of age, English-speaking, and community-dwelling. Exclusion criteria included (1) potential disease or medication confounded with fatigue symptoms: neurologic or vascular disorders (e.g., multiple sclerosis, stroke, transient ischemic attack, heart attack, or traumatic brain injury within the past five years, severe cerebrovascular disease, Parkinson’s disease); an episode of a diagnosed and active psychiatric disorder (i.e., major depression, anxiety, bipolar disorder) within the past five years; schizophrenia (regardless of the time since the last episode); a clinical diagnosis of mild cognitive impairment or dementia as defined by the most current version of DSM; change in beta-blocker dosage within the past three months; and (2) MRI contraindications (e.g., pacemaker, metallic implant, claustrophobia). The study was approved by the university’s research subject review board (IRB No. RSRB00063603). All the participants were required to understand the experimental procedure, answer UBACC correctly, and sign the informed consent.

### CF assessment

An 18-item visual analogue scale (VAS) was used to assess fatigue before and immediately after MRI scan. The VAS was adapted [33], using a Likert scale ranging from 0 (“not at all") to 10 (“very much”). Participants were instructed to rate their current state based on items related to mood and energy (e.g. tired, keeping your eyes open is difficult, lively, efficient). All “alertness” items were reversely coded and a mean score across all items was developed with higher scores indicating higher fatigue. The Cronbach’s alpha of the two measures were both >.90. CF was computed as the discrepancy between fatigue mean scores after and before MRI scan, where higher scores indicated more fatigue after the tasks. There is no standard cutoff score for defining fatigue vs. non-fatigue after performing cognitive tasks in older adults, especially since the meaning of scores can be varied depending on the type, duration, and intensity of the manipulation task. To better extract the CF related cortical-striatal network, we first selected two subgroups with distinct CF sensitivity, based on the entire sample’s CF score distribution, as the low-CF (CF≤ 0, n = 12) and high-CF (CF ≥ 1, n = 14) group (see Figure 2A). We next used the entire sample’s CF score for further analyses.

### Design and CF task protocol

An MRI related study visit occurred during a controlled morning window (8-12am) to avoid a potential diurnal fluctuation in the CF indices. Participants were instructed to eat breakfast but to avoid nicotine, caffeine, and exercise for at least 2 hours before their arrival. Participants rested for 5 minutes upon arrival, and rated pre-task fatigue using an 18-item VAS [33]. The MRI scan started with a T1-weighted sequence, followed by cognitively fatiguing task related BOLD fMRI. Another VAS rating of post-task fatigue was completed immediately after the BOLD fMRI.

To induce fatigue, the participant was required to complete two cognitive tasks in the scanner: Color-word Stroop task and Dual 1-back task (Figure 1). Event-related design was used. In the Stroop task, participants were shown serial colored words on the screen, and asked to judge the color of the word regardless of the meaning of the word. In the Dual 1-back task participants were shown an English letter on the screen and asked to judge whether the current stimulus matched the letter and position of the previous one. For both tasks, a visual stimulus can last up to 4 seconds; participants were required to make response before the stimulus disappeared. A feedback was provided for 1.5 seconds. A 3-second interval was provided between stimuli. For Stroop task, an extra 0.5-second fixation (white dot before the onset of stimulus) was placed to remind the participant of preparing for the appearance of next stimulus. For 1-back task, since the task required a comparison between stimuli, subject did not need a 0.5 second fixation for reminding. Each of the tasks lasted 15 minutes, and the order of the two tasks was randomized across participants.

### Behavioral data analysis

In the two cognitively fatiguing tasks, data on the accuracy and reaction time (RT) in response to each stimulus were collected. To assess cognitive performance, we calculated intra-individual variability of reaction time (IIVRT), using standard deviation divided by average reaction time to trials with correct responses for each task. IIVRT is considered a better measurement of cognitive function than accuracy or RT in aging [34], which ensures comparability of performance between two types of task. Before calculating IIVRT, the two cognitive tasks were first sorted based on the task order irrespective of the type. Then we divided each task into 3 blocks (5 minutes each), and calculated the IIVRT within each block. Cognitive performance throughout the protocol was calculated as the IIVRT difference between Block 6 and Block 1, with lower scores indicating better cognitive performance.

### Assessment of overall executive abilities

General cognitive performance was assessed using tests reflecting working memory (1-back with calculation as the distraction task) and cognitive control (Set shifting task) that are conceptually similar to the cognitively fatiguing tasks, but format-wise different, from the EXAMINER package [35]. Higher composite score indicated better executive function. This additional outside-MRI measure of general cognitive capacity was included to test whether the relationship between CF and PASA model can be applicable to more generalized circumstances.

### Imaging data acquisition

Imaging data were collected at the Rochester Center for Brain Imaging using a 3T Siemens TrioTIM scanner (Erlangen, Germany). The fMRI scan began with a MPRAGE scan (TR/TE = 2530/3.44 ms, TI = 1100 ms, FA = 7, matrix = 256×256, resolution 1×1×1mm, slice thickness = 1 mm, 192 slices). The task-related fMRI data were collected using a gradient echo-planar imaging (EPI) sequence (TR/TE = 2500ms/30ms, FA = 90, slice thickness = 4 mm, matrix = 64 × 64, 4×4 mm in-plane resolution, 42 axial slices, volumes = 360), lasting 15 minutes for each cognitively fatiguing task.

### Gray matter volume

Voxel-based morphometry (VBM) analysis was performed to generate a whole-brain gray matter map using SPM8. First, the structural images were segmented into gray matter, white matter and cerebrospinal ﬂuid. Second, a gray matter template was generated through an iteratively nonlinear registration using DARTEL, a toolbox with a fast diffeomorphic registration algorithm [36]. The generated gray matter template was used for normalizing functional images to MNI space. Third, the gray matter mask was applied to extract global gray matter volume from individual’s gray matter image. Since brain atrophy is a crucial factor of neurodegeneration, the whole-brain gray matter volume was used as a covariate.

### Functional imaging data preprocessing

The fMRI data were preprocessed using the Data Processing Assistant for Resting-State fMRI (DPARSFA) [37] based on SPM8 (http://www.fil.ion.ucl.ac.uk/spm/). For each participant, the first 6 volumes per task were excluded to obtain steady-state tissue magnetization. The remaining imaging data were corrected for slice timing and head-motion, co-registered to their own structural images, and normalized to the Montreal Neurological Institute (MNI) standard space. All processed fMRI images were resampled to 3×3×3 mm, and smoothed using a Gaussian kernel (FWHM 6 mm). Six subjects were excluded from the formal analysis due to low quality coregistration and head motion greater than 2 mm or 2 degrees. In addition, given the commonality of head motion in old age and its confounding effect [38], we also examined the relationship between head motion and CF, which showed no significant correlations.

### Event-related functional connectivity analysis

We applied beta-series correlation analysis, which allows the investigation of functional connectivity in event-related fMRI data [39,40]. To examine the functional change over time, the two fMRI tasks were sorted based on the order irrespective of the type. Then we divided the series of beta-values across the 30-minute protocol into 6 blocks (5 minutes each), the same time segmentation as calculating block-based IIVRT described above. A general linear model (GLM) was applied to estimate the evoked activity within a block; Block-based beta-values related to a given experimental condition was therefore generated for each voxel. To generate the cortical-striatal network, the bilateral striatum were selected as seeds based on the Automated anatomical labeling (AAL) template [41]. The individual’s cortical-striatal networks were generated using beta-series correlation between a seed and a candidate voxel in the BASCO toolbox [40]. We applied repeated-measures ANOVA to examine the interaction effects of CF subgroup (low vs. high) by task block (block1 vs. block6) on the block-based beta-series correlations between the seeds (striatum at each hemisphere) and candidate voxels (whole brain except the striatum being used as the seed), to identify the functional connectivity with significant changes over blocks that differed between CF subgroups. Statistical F maps were generated with threshold at p < 0.05 by GRF correction (individual p < 0.005, cluster p < 0.05).

### PASA model

To examine the PASA model in CF related cortical-striatal network, the averaged strength of functional connectivity was calculated, based on beta-series correlations, across regions for anterior and posterior brain respectively, regardless of hemisphere. Posterior-anterior shift (PAS) score was defined as

PAS score = Block6(Anterior – Posterior) – Block1(Anterior – Posterior)

to estimate the shifting degree from posterior to anterior regions with higher scores indicating greater reliance on anterior regions at later time points.

### Other statistical analysis

All other statistical analyses were conducted using SPSS 22.0 (IBM Corporation, Armonk, NY). Non-parametric group comparison was conducted for the comparison of high- vs. low-CF subgroups. We also used repeated measures ANOVA to examine the interaction effect between tasks (pre- vs. post-) and groups (high- vs. low-CF) on CF, as well as the interaction effect between task blocks (1 vs. 6) and groups (high- vs. low-CF) on IIVRT. Partial correlation was applied to examine relationships between CF, cortical-striatal networks, and task performance, controlling for age, education and whole-brain gray matter volume. Moderation analysis was used to test whether CF would moderate the relationship between PAS score and cognitive performance using macro PROCESS (http://www.processmacro.org), controlling for age, education and whole-brain gray matter volume.

## Supplementary Material

Supplementary Figures

Supplementary Table
