# Supplementary material for: Cognitive fatigue and cortical-striatal network in old age
Source: Aging (Albany NY). 2019 Apr 17;11(8):2312–26. doi: 10.18632/aging.101915 (PMC6519999; doi:10.18632/aging.101915)
Supplement: Supplementary Figures [file aging-11-101915-s001.pdf]

SUPPLEMENTARY TABLE

Supplementary Table 1. Demographics and clinical characteristics in low- and high-CF group.

	Low-CF (n = 12)	High-CF (n = 14)	Mann-Whitney U or χ^2 test (p value)
Age, Median (IQR)	72.50 (6.50)	69.50 (7.75)	.63
Male, n (%)	5 (41.6)	3 (21.4)	.27
Years of education, Median (IQR)	18.00 (7.00)	16.00 (2.00)	.78
MOCA, Median (IQR)	29.00 (2.50)	28.50 (2.50)	.86
CF-pre, Median (IQR)	1.90 (0.75)	1.11 (1.49)	.085
CF change, Median (IQR)	-0.35 (0.97)	3.05 (1.86)	< **.001**
IIVRT change, Median (IQR)	-0.018 (0.13)	0.016 (0.12)	.085
Brain atrophy, Median (IQR)	0.36 (0.05)	0.34 (0.03)	.23
Executive abilities, Median (IQR)	0.58 (0.38)	0.70 (0.51)	1.0

Note. MOCA, Montreal Cognitive Assessment; CF, cognitive fatigue; IIVRT, intra-individual variability of reaction time; SD, standard deviation; IQR, interquartile range.
