# Supplementary material for: Cognitive fatigue and cortical-striatal network in old age
Source: Aging (Albany NY). 2019 Apr 17;11(8):2312–26. doi: 10.18632/aging.101915 (PMC6519999; doi:10.18632/aging.101915)
Supplement: Supplementary Table [file aging-11-101915-s002.pdf]

## SUPPLEMENTARY FIGURES

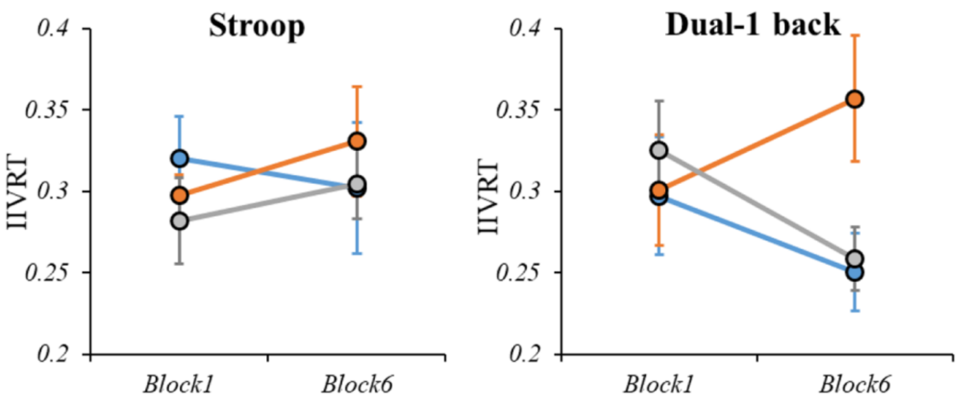


**Supplementary Figure 1. IIVRT across task blocks in Stroop and Dual-1 back tasks.** In Stroop task, there was a marginal significant subgroup (high- vs. low-CF) by block (1 vs. 6) interaction on IIVRT ($F(1, 11) = 4.34$, $p = .061$). In Dual-1back task, there was no significant subgroup (high- vs. low-CF) by block (1 vs. 6) interaction on IIVRT ($F(1, 11) = 0.38$, $p = .55$). Although there was no significant interaction effect within each task, the IIVRT change of low- and high-CF was comparable between the two tasks. Note: CF, cognitive fatigue; IIVRT, intra-individual variability of reaction time.

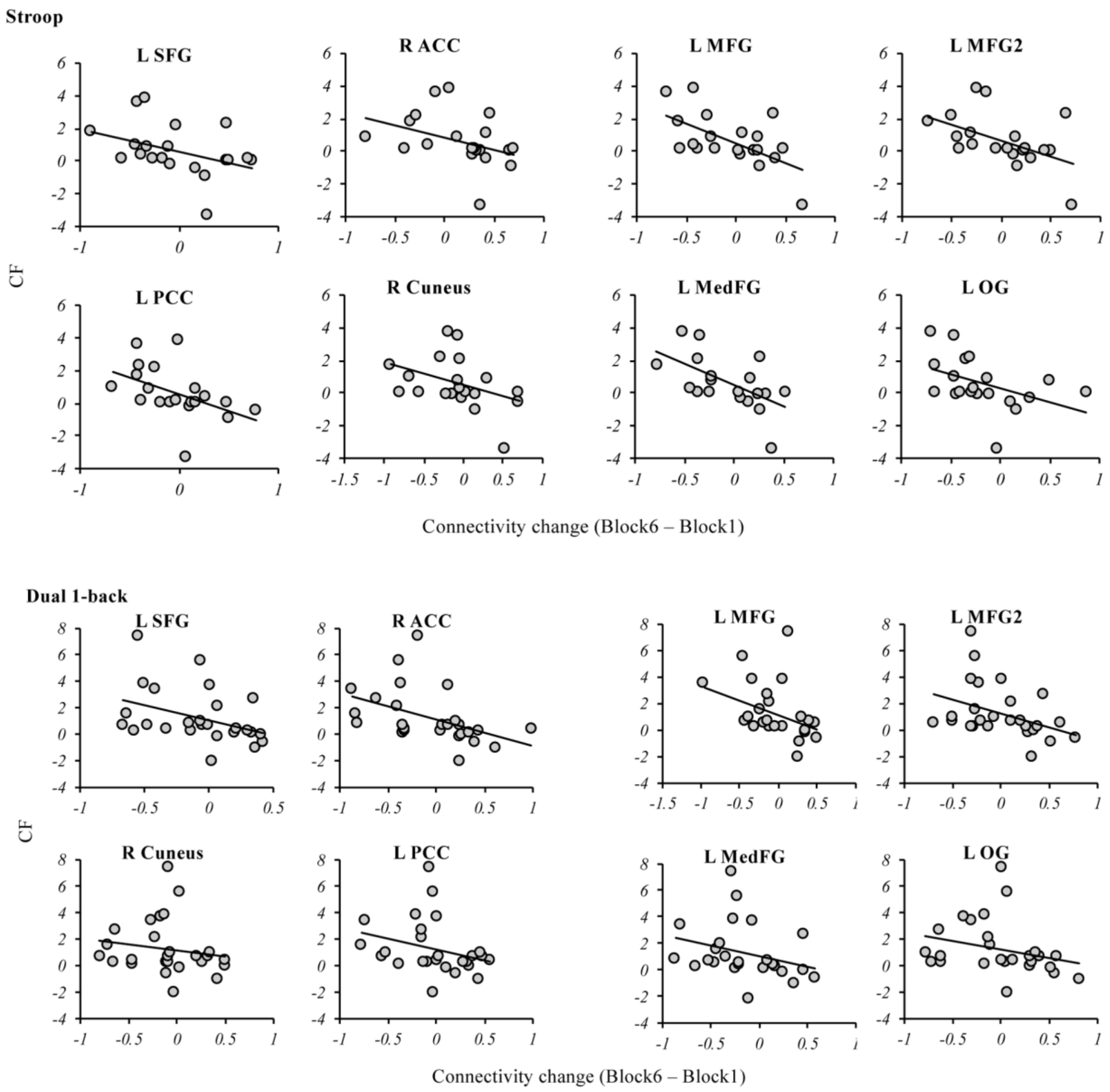


**Supplementary Figure 2. Correlations between the cortical-striatal network and CF in Stroop and Dual-1 back tasks.** The consistent negative trend was found in correlations between CF change and connectivity strength across the two tasks. Note: L, left; R, right; SFG, superior frontal gyrus; ACC, anterior cingulate cortex; PCC, posterior cingulate cortex; MedFG, medial frontal gyrus, MFG, middle frontal gyrus; OG, occipital gyrus. CF, cognitive fatigue.

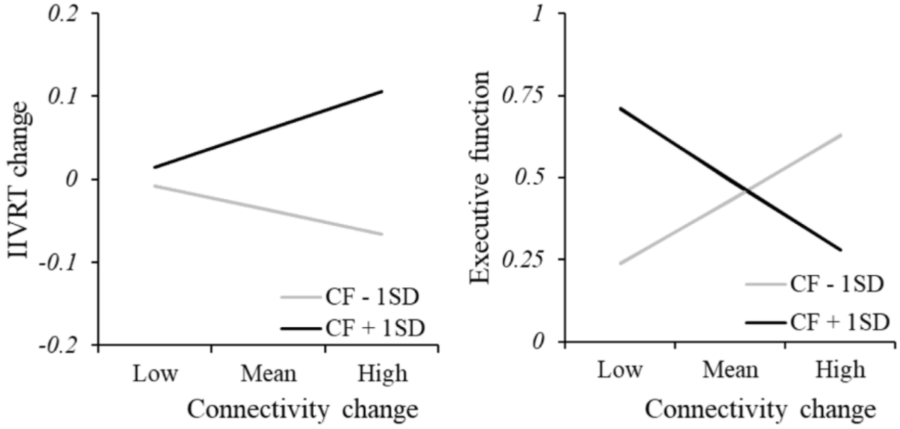


**Supplementary Figure 3. Moderation analysis in relationship between anterior striatal network and cognitive function.** There was a significant moderating effect of CF change on anterior network and IIVRT change/executive function. The results were consistent with the findings in moderation analysis in PAS. Note: CF, cognitive fatigue; IIVRT, intra-individual variability of reaction time. PAS, posterior-anterior shifting.
